# Bis(1*H*-benzimidazole-2-carboxyl­ato-κ^2^ *N* ^3^,*O*)bis­(ethanol-κ*O*)manganese(II)

**DOI:** 10.1107/S1600536812039475

**Published:** 2012-09-22

**Authors:** Jian-Hua Nie, Yue-Hua Lin, Chun-Tao Xu

**Affiliations:** a Zhongshan Polytechnic, Zhongshan, Guangdong 528404, People’s Republic of China

## Abstract

In the title compound, [Mn(C_8_H_5_N_2_O_2_)_2_(C_2_H_5_OH)_2_], the Mn^II^ atom is six-coordinated by two N and two O atoms from two 1*H*-benzimidazole-2-carboxyl­ate (*L*) ligands and by two O atoms from two ethanol mol­ecules in a distorted octa­hedral geometry. The mean planes of the two *L* ligands are inclined to each other at 7.6 (1)°. In the crystal, N—H⋯O and O—H⋯O hydrogen bonds link the mol­ecules into layers parallel to the *ab* plane.

## Related literature

For related structures, see: Carballo *et al.* (1996 ▶); Di *et al.* (2010 ▶); Fan *et al.* (2011 ▶); Małecki & Maroń (2012 ▶); Rettig *et al.* (1999 ▶); Saczewski *et al.* (2006 ▶); Zheng *et al.* (2011 ▶).
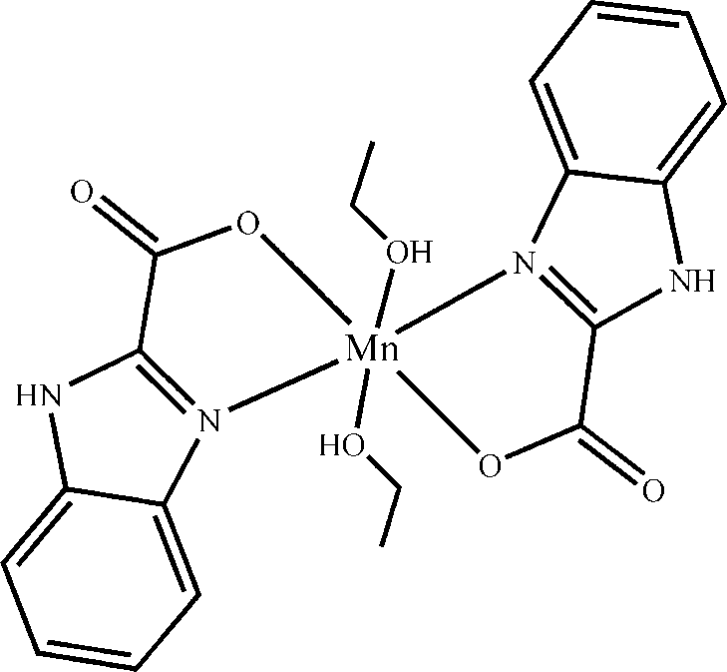


## Experimental

### 

#### Crystal data

[Mn(C_8_H_5_N_2_O_2_)_2_(C_2_H_6_O)_2_]
*M* *_r_* = 469.36
Triclinic, 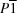

*a* = 5.4176 (12) Å
*b* = 10.358 (2) Å
*c* = 19.853 (5) Å
α = 75.671 (3)°
β = 88.294 (3)°
γ = 78.230 (3)°
*V* = 1056.4 (4) Å^3^
*Z* = 2
Mo *K*α radiation
μ = 0.67 mm^−1^
*T* = 298 K
0.32 × 0.25 × 0.22 mm

#### Data collection

Bruker APEXII CCD diffractometer
Absorption correction: multi-scan (*SADABS*; Bruker, 2004 ▶) *T*_min_ = 0.814, *T*_max_ = 0.867
5431 measured reflections
3751 independent reflections
2664 reflections with *I* > 2σ(*I*)
*R*_int_ = 0.022

#### Refinement

*R*[*F* ^2^ > 2σ(*F* ^2^)] = 0.048
*wR*(*F* ^2^) = 0.131
*S* = 1.06
3751 reflections
282 parameters
H-atom parameters constrained
Δρ_max_ = 0.40 e Å^−3^
Δρ_min_ = −0.37 e Å^−3^

### 

Data collection: *APEX2* (Bruker, 2004 ▶); cell refinement: *SAINT* (Bruker, 2004 ▶); data reduction: *SAINT*; program(s) used to solve structure: *SHELXS97* (Sheldrick, 2008 ▶); program(s) used to refine structure: *SHELXL97* (Sheldrick, 2008 ▶); molecular graphics: *ORTEPIII* (Burnett & Johnson, 1996 ▶) and *PLATON* (Spek, 2009 ▶); software used to prepare material for publication: *SHELXL97*.

## Supplementary Material

Crystal structure: contains datablock(s) I, global. DOI: 10.1107/S1600536812039475/cv5342sup1.cif

Structure factors: contains datablock(s) I. DOI: 10.1107/S1600536812039475/cv5342Isup2.hkl

Additional supplementary materials:  crystallographic information; 3D view; checkCIF report

## Figures and Tables

**Table 1:** Hydrogen-bond geometry (Å, °)

*D*—H⋯*A*	*D*—H	H⋯*A*	*D*⋯*A*	*D*—H⋯*A*
N2—H2⋯O4^i^	0.86	1.96	2.766 (3)	155
N4—H4⋯O2^ii^	0.86	1.97	2.786 (3)	158
O5—H5*A*⋯O3^iii^	0.85	1.89	2.710 (3)	161
O6—H6*A*⋯O1^iv^	0.85	1.88	2.692 (3)	158

Symmetry codes: (i) 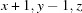; (ii) 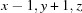; (iii) 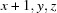; (iv) 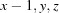.

**Fig. 1.**
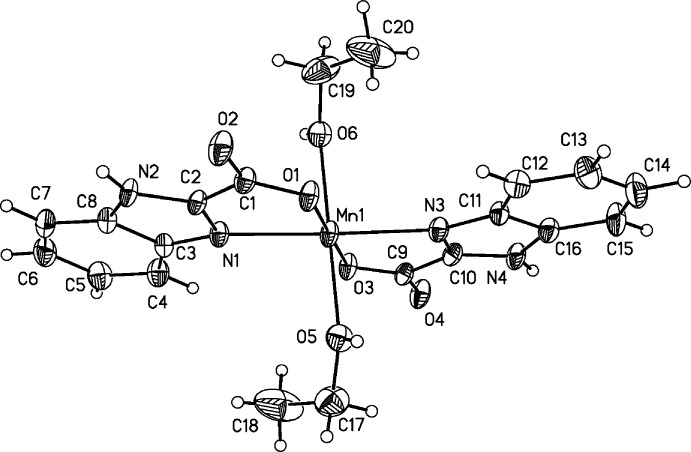
The molecular structure of the title compound, with displacement ellipsoids drawn at the 30% probability level.

**Fig. 2.**
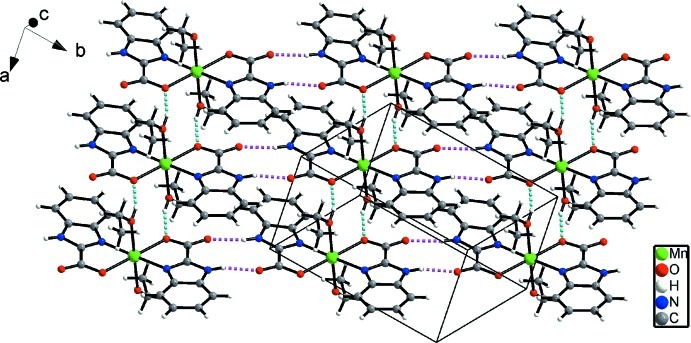
The crystal packing of the title compound, showing the two-dimensional supramolecular network. Hydrogen bonds are shown as dashed lines.

## supplementary crystallographic information

### Comment

N-Heterocyclic carboxylic acids, a kind of multidentate ligands for the construction of new metal coordination polymers, have attracted much attention not only because their versatile coordination behaviors but also owing to their forming high-dimensional polymers through hydrogen-bonding interactions in the process of self-assembly. 1*H*-benzimidazole-2-carboxylic acid (H*L*), which includes two nitrogen atoms of an aromatic group and one carboxylate group, is an ideal candidate for preparing new coordination polymers. Up to now, several coordination polymers with low-dimensional structural features based on the H*L* ligand have been investigated (Carballo *et al.*, 1996; Di *et al.*, 2010; Fan *et al.*, 2011; Małecki & Maroń, 2012; Rettig *et al.*, 1999; Saczewski *et al.*, 2006; Zheng *et al.*, 2011). For example, Fan *et al.* (2011) have described the structure of a mononuclear complex [Cd(*L*)_2_(C_2_H_5_OH)_2_]. In this paper, we report a new Mn^II^ coordination polymer [Mn(*L*)_2_(C_2_H_5_OH)_2_], which is isomorphous with the Cd^II^ analog.

The asymmetric unit of the title compound contains one Mn^II^ ion, two *L* anions and two coordinated ethanol molecules. As illustrated in Fig. 1, the Mn^II^ ion is six-coordinated with two N and two O atoms from two bidentate chelating *L* ligands in the equatorial plane, and two ethanol molecules in axial positions, forming a slightly distorted octahedral geometry. The Mn—N bond lengths are in the range of 2.227 (2)–2.230 (2) Å, and the Mn—O distances vary from 2.197 (2) to 2.235 (2) Å, all of which are similar to those in Cd^II^ analog. In the crystal structure, pairs of intermolecular N—H···O hydrogen bonds (Table1) link the molecules into one-dimensional chains, which are further connected by O—H···O hydrogen bonds involving the carboxylate O atoms of the *L* ligands and the coordinated ethanol molecules, resulting in the formation of a two-dimensional supramolecular network (Fig. 2).

### Experimental

A mixture of H*L* (0.30 mmol), MnCl_2_ (0.30 mmol) and 8 ml C_2_H_5_OH was sealed into a 15 ml Teflon-lined stainless steel autoclave, heated at 393 K for 48 h under autogenous pressure, and then slowly cooled to room temperature at a rate of 5 k /h. Pink block crystals of the title compound were obtained, washed with distilled water, and dried in air (yield: 38%).

### Refinement

C- and N-bound H atoms were positioned geometrically and refined using a riding model, with C—H = 0.93–0.97 Å, N—H = 0.86 Å and with *U*_iso_(H) = 1.2(1.5 for methyl)*U*_eq_(C), *U*_iso_(H) = 1.2*U*_eq_(N). Hydroxy H atoms were located in a difference Fourier map and refined as riding atoms, with O—H = 0.85 Å and *U*_iso_(H) = 1.2*U*_eq_(O).

### Crystal data

**Table:**

[Mn(C_8_H_5_N_2_O_2_)_2_(C_2_H_6_O)_2_]	*Z* = 2
*M**_r_* = 469.36	*F*(000) = 486
Triclinic, *P*1	*D*_x_ = 1.476 Mg m^−^^3^
Hall symbol: -P 1	Mo *K*α radiation, λ = 0.71073 Å
*a* = 5.4176 (12) Å	Cell parameters from 1379 reflections
*b* = 10.358 (2) Å	θ = 2.6–26.0°
*c* = 19.853 (5) Å	µ = 0.67 mm^−^^1^
α = 75.671 (3)°	*T* = 298 K
β = 88.294 (3)°	Block, pink
γ = 78.230 (3)°	0.32 × 0.25 × 0.22 mm
*V* = 1056.4 (4) Å^3^	

### Data collection

**Table:**

Bruker APEXII CCD diffractometer	3751 independent reflections
Radiation source: fine-focus sealed tube	2664 reflections with *I* > 2σ(*I*)
Graphite monochromator	*R*_int_ = 0.022
φ and ω scans	θ_max_ = 25.3°, θ_min_ = 2.1°
Absorption correction: multi-scan (*APEX2*; Bruker, 2004)	*h* = −6→6
*T*_min_ = 0.814, *T*_max_ = 0.867	*k* = −12→12
5431 measured reflections	*l* = −23→19

### Refinement

**Table:**

Refinement on *F*^2^	Primary atom site location: structure-invariant direct methods
Least-squares matrix: full	Secondary atom site location: difference Fourier map
*R*[*F*^2^ > 2σ(*F*^2^)] = 0.048	Hydrogen site location: inferred from neighbouring sites
*wR*(*F*^2^) = 0.131	H-atom parameters constrained
*S* = 1.06	*w* = 1/[σ^2^(*F*_o_^2^) + (0.0642*P*)^2^ + 0.1644*P*] where *P* = (*F*_o_^2^ + 2*F*_c_^2^)/3
3751 reflections	(Δ/σ)_max_ < 0.001
282 parameters	Δρ_max_ = 0.40 e Å^−^^3^
0 restraints	Δρ_min_ = −0.37 e Å^−^^3^

### Special details

**Table:**

Geometry. All e.s.d.'s (except the e.s.d. in the dihedral angle between two l.s. planes) are estimated using the full covariance matrix. The cell e.s.d.'s are taken into account individually in the estimation of e.s.d.'s in distances, angles and torsion angles; correlations between e.s.d.'s in cell parameters are only used when they are defined by crystal symmetry. An approximate (isotropic) treatment of cell e.s.d.'s is used for estimating e.s.d.'s involving l.s. planes.
Refinement. Refinement of *F*^2^ against ALL reflections. The weighted *R*-factor *wR* and goodness of fit *S* are based on *F*^2^, conventional *R*-factors *R* are based on *F*, with *F* set to zero for negative *F*^2^. The threshold expression of *F*^2^ > σ(*F*^2^) is used only for calculating *R*-factors(gt) *etc*. and is not relevant to the choice of reflections for refinement. *R*-factors based on *F*^2^ are statistically about twice as large as those based on *F*, and *R*-factors based on ALL data will be even larger.

### Fractional atomic coordinates and isotropic or equivalent isotropic displacement parameters (Å^2^)

**Table:**

	*x*	*y*	*z*	*U*_iso_*/*U*_eq_	
Mn1	0.46488 (9)	0.05915 (4)	0.74701 (2)	0.03348 (18)	
O1	0.8162 (4)	−0.08678 (19)	0.78515 (11)	0.0369 (5)	
O6	0.2473 (4)	−0.0469 (2)	0.83306 (11)	0.0422 (6)	
H6A	0.0932	−0.0433	0.8233	0.051*	
O5	0.6780 (4)	0.1657 (2)	0.66112 (11)	0.0461 (6)	
H5A	0.8299	0.1625	0.6730	0.055*	
O3	0.1113 (4)	0.2050 (2)	0.71013 (11)	0.0368 (5)	
O2	1.0056 (4)	−0.3035 (2)	0.79681 (12)	0.0481 (6)	
O4	−0.0995 (4)	0.4137 (2)	0.70807 (12)	0.0476 (6)	
N4	0.2721 (5)	0.4332 (2)	0.80378 (14)	0.0402 (7)	
H4	0.1633	0.5087	0.7945	0.048*	
N1	0.4569 (5)	−0.1113 (2)	0.69784 (13)	0.0333 (6)	
N3	0.4673 (5)	0.2256 (2)	0.79975 (13)	0.0334 (6)	
N2	0.6283 (5)	−0.3281 (2)	0.70541 (13)	0.0376 (7)	
H2	0.7302	−0.4056	0.7181	0.045*	
C3	0.3176 (6)	−0.1605 (3)	0.65648 (15)	0.0329 (7)	
C9	0.0768 (6)	0.3172 (3)	0.72763 (16)	0.0331 (7)	
C8	0.4246 (6)	−0.2976 (3)	0.66079 (16)	0.0355 (8)	
C16	0.4743 (6)	0.4005 (3)	0.84869 (17)	0.0380 (8)	
C2	0.6400 (6)	−0.2151 (3)	0.72576 (16)	0.0322 (7)	
C12	0.8195 (7)	0.2074 (3)	0.88379 (17)	0.0438 (8)	
H12	0.9051	0.1210	0.8818	0.053*	
C7	0.3212 (7)	−0.3756 (3)	0.62573 (18)	0.0473 (9)	
H7	0.3924	−0.4668	0.6295	0.057*	
C15	0.5634 (8)	0.4703 (4)	0.89100 (19)	0.0552 (10)	
H15	0.4793	0.5567	0.8934	0.066*	
C10	0.2738 (6)	0.3254 (3)	0.77646 (16)	0.0337 (7)	
C11	0.5984 (6)	0.2698 (3)	0.84512 (16)	0.0327 (7)	
C4	0.1007 (7)	−0.0984 (3)	0.61532 (18)	0.0456 (9)	
H4A	0.0262	−0.0077	0.6119	0.055*	
C5	0.0011 (7)	−0.1746 (4)	0.58016 (18)	0.0505 (9)	
H5	−0.1421	−0.1344	0.5521	0.061*	
C1	0.8394 (6)	−0.2038 (3)	0.77318 (16)	0.0342 (7)	
C6	0.1096 (8)	−0.3117 (4)	0.58541 (19)	0.0544 (10)	
H6	0.0361	−0.3605	0.5609	0.065*	
C13	0.9074 (7)	0.2778 (4)	0.92515 (19)	0.0548 (10)	
H13	1.0557	0.2384	0.9511	0.066*	
C14	0.7791 (8)	0.4069 (4)	0.9290 (2)	0.0627 (12)	
H14	0.8422	0.4509	0.9581	0.075*	
C17	0.6190 (9)	0.2498 (4)	0.5940 (2)	0.0713 (12)	
H17A	0.5509	0.3418	0.5977	0.086*	
H17B	0.7733	0.2512	0.5680	0.086*	
C19	0.3144 (9)	−0.1405 (5)	0.8966 (2)	0.0818 (15)	
H19A	0.1628	−0.1460	0.9235	0.098*	
H19B	0.3765	−0.2291	0.8875	0.098*	
C18	0.4453 (11)	0.2095 (7)	0.5564 (2)	0.124 (2)	
H18A	0.2976	0.1994	0.5835	0.187*	
H18B	0.5205	0.1242	0.5463	0.187*	
H18C	0.3990	0.2773	0.5137	0.187*	
C20	0.4932 (11)	−0.1153 (7)	0.9374 (2)	0.123 (2)	
H20A	0.5437	−0.1930	0.9757	0.185*	
H20B	0.4227	−0.0373	0.9547	0.185*	
H20C	0.6374	−0.0982	0.9099	0.185*	

### Atomic displacement parameters (Å^2^)

**Table:**

	*U*^11^	*U*^22^	*U*^33^	*U*^12^	*U*^13^	*U*^23^
Mn1	0.0294 (3)	0.0244 (3)	0.0481 (3)	0.00030 (19)	−0.0057 (2)	−0.0157 (2)
O1	0.0298 (13)	0.0262 (11)	0.0574 (14)	0.0004 (9)	−0.0091 (10)	−0.0188 (10)
O6	0.0336 (13)	0.0479 (13)	0.0476 (14)	−0.0115 (11)	−0.0036 (10)	−0.0131 (11)
O5	0.0371 (14)	0.0578 (15)	0.0456 (14)	−0.0137 (11)	−0.0057 (11)	−0.0127 (12)
O3	0.0317 (13)	0.0281 (11)	0.0536 (14)	−0.0012 (9)	−0.0084 (10)	−0.0181 (10)
O2	0.0421 (15)	0.0307 (12)	0.0695 (16)	0.0061 (11)	−0.0190 (12)	−0.0174 (11)
O4	0.0412 (15)	0.0268 (12)	0.0727 (17)	0.0047 (11)	−0.0160 (12)	−0.0157 (11)
N4	0.0433 (18)	0.0220 (13)	0.0554 (17)	0.0011 (12)	−0.0068 (14)	−0.0150 (12)
N1	0.0303 (16)	0.0270 (13)	0.0434 (15)	0.0008 (11)	−0.0032 (12)	−0.0150 (12)
N3	0.0307 (15)	0.0263 (13)	0.0433 (15)	−0.0005 (11)	−0.0031 (12)	−0.0126 (11)
N2	0.0399 (17)	0.0221 (13)	0.0530 (17)	−0.0018 (12)	−0.0056 (13)	−0.0162 (12)
C3	0.0326 (19)	0.0312 (16)	0.0380 (18)	−0.0054 (14)	−0.0002 (14)	−0.0149 (14)
C9	0.0323 (19)	0.0228 (15)	0.0436 (18)	−0.0016 (14)	0.0002 (14)	−0.0104 (14)
C8	0.0336 (19)	0.0331 (17)	0.0435 (19)	−0.0053 (14)	−0.0004 (15)	−0.0174 (14)
C16	0.040 (2)	0.0300 (17)	0.0450 (19)	−0.0051 (15)	−0.0039 (16)	−0.0119 (15)
C2	0.0335 (19)	0.0209 (15)	0.0425 (18)	−0.0014 (13)	−0.0013 (14)	−0.0110 (13)
C12	0.038 (2)	0.0433 (19)	0.049 (2)	−0.0049 (16)	−0.0007 (16)	−0.0127 (16)
C7	0.055 (3)	0.0373 (19)	0.056 (2)	−0.0115 (17)	0.0024 (19)	−0.0234 (17)
C15	0.064 (3)	0.042 (2)	0.069 (3)	−0.0064 (19)	−0.010 (2)	−0.0322 (19)
C10	0.0329 (19)	0.0226 (15)	0.0449 (19)	−0.0029 (13)	0.0014 (14)	−0.0096 (13)
C11	0.0350 (19)	0.0278 (16)	0.0371 (17)	−0.0052 (14)	−0.0031 (14)	−0.0122 (13)
C4	0.039 (2)	0.0414 (19)	0.057 (2)	0.0008 (16)	−0.0056 (17)	−0.0199 (17)
C5	0.042 (2)	0.059 (2)	0.053 (2)	−0.0095 (18)	−0.0128 (18)	−0.0189 (19)
C1	0.0315 (19)	0.0249 (16)	0.0449 (19)	0.0005 (14)	−0.0025 (15)	−0.0110 (14)
C6	0.060 (3)	0.059 (2)	0.056 (2)	−0.023 (2)	−0.006 (2)	−0.026 (2)
C13	0.046 (2)	0.067 (3)	0.056 (2)	−0.014 (2)	−0.0121 (18)	−0.020 (2)
C14	0.071 (3)	0.062 (3)	0.067 (3)	−0.018 (2)	−0.017 (2)	−0.031 (2)
C17	0.074 (3)	0.069 (3)	0.062 (3)	−0.014 (2)	−0.018 (2)	0.002 (2)
C19	0.079 (3)	0.070 (3)	0.081 (3)	−0.028 (3)	−0.036 (3)	0.022 (2)
C18	0.105 (5)	0.218 (7)	0.059 (3)	−0.087 (5)	−0.022 (3)	−0.004 (4)
C20	0.111 (5)	0.208 (7)	0.058 (3)	−0.090 (5)	−0.027 (3)	0.005 (4)

### Geometric parameters (Å, º)

**Table:**

Mn1—O1	2.197 (2)	C16—C11	1.402 (4)
Mn1—O3	2.201 (2)	C2—C1	1.494 (4)
Mn1—O5	2.220 (2)	C12—C13	1.376 (5)
Mn1—N1	2.227 (2)	C12—C11	1.391 (4)
Mn1—N3	2.230 (2)	C12—H12	0.9300
Mn1—O6	2.235 (2)	C7—C6	1.369 (5)
O1—C1	1.273 (3)	C7—H7	0.9300
O6—C19	1.393 (4)	C15—C14	1.363 (5)
O6—H6A	0.8535	C15—H15	0.9300
O5—C17	1.405 (4)	C4—C5	1.368 (5)
O5—H5A	0.8546	C4—H4A	0.9300
O3—C9	1.271 (3)	C5—C6	1.401 (5)
O2—C1	1.226 (3)	C5—H5	0.9300
O4—C9	1.226 (3)	C6—H6	0.9300
N4—C10	1.355 (4)	C13—C14	1.395 (5)
N4—C16	1.366 (4)	C13—H13	0.9300
N4—H4	0.8600	C14—H14	0.9300
N1—C2	1.323 (4)	C17—C18	1.404 (6)
N1—C3	1.380 (4)	C17—H17A	0.9700
N3—C10	1.316 (4)	C17—H17B	0.9700
N3—C11	1.378 (4)	C19—C20	1.384 (6)
N2—C2	1.343 (4)	C19—H19A	0.9700
N2—C8	1.371 (4)	C19—H19B	0.9700
N2—H2	0.8600	C18—H18A	0.9600
C3—C4	1.397 (4)	C18—H18B	0.9600
C3—C8	1.403 (4)	C18—H18C	0.9600
C9—C10	1.492 (4)	C20—H20A	0.9600
C8—C7	1.392 (5)	C20—H20B	0.9600
C16—C15	1.388 (4)	C20—H20C	0.9600
			
O1—Mn1—O3	179.29 (8)	C6—C7—H7	121.6
O1—Mn1—O5	89.01 (8)	C8—C7—H7	121.6
O3—Mn1—O5	91.60 (8)	C14—C15—C16	117.3 (3)
O1—Mn1—N1	76.37 (8)	C14—C15—H15	121.3
O3—Mn1—N1	103.94 (8)	C16—C15—H15	121.3
O5—Mn1—N1	93.63 (9)	N3—C10—N4	112.0 (3)
O1—Mn1—N3	103.36 (8)	N3—C10—C9	123.3 (3)
O3—Mn1—N3	76.30 (8)	N4—C10—C9	124.7 (3)
O5—Mn1—N3	88.43 (9)	N3—C11—C12	131.0 (3)
N1—Mn1—N3	177.92 (9)	N3—C11—C16	109.1 (3)
O1—Mn1—O6	91.49 (8)	C12—C11—C16	119.9 (3)
O3—Mn1—O6	87.90 (8)	C5—C4—C3	118.1 (3)
O5—Mn1—O6	179.50 (8)	C5—C4—H4A	120.9
N1—Mn1—O6	86.45 (9)	C3—C4—H4A	120.9
N3—Mn1—O6	91.50 (9)	C4—C5—C6	121.6 (3)
C1—O1—Mn1	116.15 (19)	C4—C5—H5	119.2
C19—O6—Mn1	134.0 (3)	C6—C5—H5	119.2
C19—O6—H6A	109.2	O2—C1—O1	126.1 (3)
Mn1—O6—H6A	115.6	O2—C1—C2	119.4 (3)
C17—O5—Mn1	135.8 (2)	O1—C1—C2	114.5 (2)
C17—O5—H5A	111.0	C7—C6—C5	121.6 (3)
Mn1—O5—H5A	112.8	C7—C6—H6	119.2
C9—O3—Mn1	116.57 (19)	C5—C6—H6	119.2
C10—N4—C16	107.7 (3)	C12—C13—C14	121.5 (4)
C10—N4—H4	126.1	C12—C13—H13	119.3
C16—N4—H4	126.1	C14—C13—H13	119.3
C2—N1—C3	105.3 (2)	C15—C14—C13	121.6 (4)
C2—N1—Mn1	108.91 (19)	C15—C14—H14	119.2
C3—N1—Mn1	144.9 (2)	C13—C14—H14	119.2
C10—N3—C11	105.9 (2)	C18—C17—O5	114.3 (4)
C10—N3—Mn1	109.0 (2)	C18—C17—H17A	108.7
C11—N3—Mn1	145.0 (2)	O5—C17—H17A	108.7
C2—N2—C8	107.6 (2)	C18—C17—H17B	108.7
C2—N2—H2	126.2	O5—C17—H17B	108.7
C8—N2—H2	126.2	H17A—C17—H17B	107.6
N1—C3—C4	131.3 (3)	C20—C19—O6	117.2 (4)
N1—C3—C8	109.2 (3)	C20—C19—H19A	108.0
C4—C3—C8	119.5 (3)	O6—C19—H19A	108.0
O4—C9—O3	125.6 (3)	C20—C19—H19B	108.0
O4—C9—C10	120.1 (3)	O6—C19—H19B	108.0
O3—C9—C10	114.3 (3)	H19A—C19—H19B	107.2
N2—C8—C7	132.4 (3)	C17—C18—H18A	109.5
N2—C8—C3	105.3 (3)	C17—C18—H18B	109.5
C7—C8—C3	122.3 (3)	H18A—C18—H18B	109.5
N4—C16—C15	132.9 (3)	C17—C18—H18C	109.5
N4—C16—C11	105.3 (3)	H18A—C18—H18C	109.5
C15—C16—C11	121.8 (3)	H18B—C18—H18C	109.5
N1—C2—N2	112.6 (3)	C19—C20—H20A	109.5
N1—C2—C1	122.6 (2)	C19—C20—H20B	109.5
N2—C2—C1	124.8 (3)	H20A—C20—H20B	109.5
C13—C12—C11	117.8 (3)	C19—C20—H20C	109.5
C13—C12—H12	121.1	H20A—C20—H20C	109.5
C11—C12—H12	121.1	H20B—C20—H20C	109.5
C6—C7—C8	116.9 (3)		
			
O5—Mn1—O1—C1	−104.5 (2)	C3—N1—C2—N2	−0.1 (4)
N1—Mn1—O1—C1	−10.5 (2)	Mn1—N1—C2—N2	172.0 (2)
N3—Mn1—O1—C1	167.4 (2)	C3—N1—C2—C1	178.4 (3)
O6—Mn1—O1—C1	75.5 (2)	Mn1—N1—C2—C1	−9.5 (4)
O1—Mn1—O6—C19	16.9 (3)	C8—N2—C2—N1	0.2 (4)
O3—Mn1—O6—C19	−162.8 (3)	C8—N2—C2—C1	−178.3 (3)
N1—Mn1—O6—C19	93.1 (3)	N2—C8—C7—C6	178.8 (4)
N3—Mn1—O6—C19	−86.5 (3)	C3—C8—C7—C6	0.9 (5)
O1—Mn1—O5—C17	155.4 (3)	N4—C16—C15—C14	−178.6 (4)
O3—Mn1—O5—C17	−24.9 (3)	C11—C16—C15—C14	0.5 (6)
N1—Mn1—O5—C17	79.1 (3)	C11—N3—C10—N4	0.5 (4)
N3—Mn1—O5—C17	−101.2 (3)	Mn1—N3—C10—N4	−176.7 (2)
O5—Mn1—O3—C9	−81.6 (2)	C11—N3—C10—C9	−177.6 (3)
N1—Mn1—O3—C9	−175.8 (2)	Mn1—N3—C10—C9	5.3 (4)
N3—Mn1—O3—C9	6.4 (2)	C16—N4—C10—N3	−1.2 (4)
O6—Mn1—O3—C9	98.4 (2)	C16—N4—C10—C9	176.8 (3)
O1—Mn1—N1—C2	9.8 (2)	O4—C9—C10—N3	179.7 (3)
O3—Mn1—N1—C2	−169.5 (2)	O3—C9—C10—N3	0.0 (5)
O5—Mn1—N1—C2	97.9 (2)	O4—C9—C10—N4	1.9 (5)
O6—Mn1—N1—C2	−82.6 (2)	O3—C9—C10—N4	−177.9 (3)
O1—Mn1—N1—C3	176.6 (4)	C10—N3—C11—C12	−178.8 (3)
O3—Mn1—N1—C3	−2.8 (4)	Mn1—N3—C11—C12	−3.6 (6)
O5—Mn1—N1—C3	−95.3 (4)	C10—N3—C11—C16	0.5 (4)
O6—Mn1—N1—C3	84.2 (4)	Mn1—N3—C11—C16	175.7 (3)
O1—Mn1—N3—C10	175.0 (2)	C13—C12—C11—N3	179.8 (3)
O3—Mn1—N3—C10	−5.7 (2)	C13—C12—C11—C16	0.5 (5)
O5—Mn1—N3—C10	86.4 (2)	N4—C16—C11—N3	−1.2 (4)
O6—Mn1—N3—C10	−93.2 (2)	C15—C16—C11—N3	179.5 (3)
O1—Mn1—N3—C11	−0.2 (4)	N4—C16—C11—C12	178.2 (3)
O3—Mn1—N3—C11	179.1 (4)	C15—C16—C11—C12	−1.1 (5)
O5—Mn1—N3—C11	−88.8 (4)	N1—C3—C4—C5	−179.1 (3)
O6—Mn1—N3—C11	91.7 (4)	C8—C3—C4—C5	−0.4 (5)
C2—N1—C3—C4	178.9 (3)	C3—C4—C5—C6	0.8 (6)
Mn1—N1—C3—C4	11.9 (6)	Mn1—O1—C1—O2	−171.0 (3)
C2—N1—C3—C8	0.0 (3)	Mn1—O1—C1—C2	8.8 (3)
Mn1—N1—C3—C8	−167.0 (3)	N1—C2—C1—O2	−179.3 (3)
Mn1—O3—C9—O4	174.7 (3)	N2—C2—C1—O2	−0.9 (5)
Mn1—O3—C9—C10	−5.6 (3)	N1—C2—C1—O1	0.9 (4)
C2—N2—C8—C7	−178.4 (4)	N2—C2—C1—O1	179.3 (3)
C2—N2—C8—C3	−0.2 (4)	C8—C7—C6—C5	−0.5 (6)
N1—C3—C8—N2	0.1 (4)	C4—C5—C6—C7	−0.3 (6)
C4—C3—C8—N2	−178.9 (3)	C11—C12—C13—C14	0.5 (6)
N1—C3—C8—C7	178.5 (3)	C16—C15—C14—C13	0.6 (6)
C4—C3—C8—C7	−0.5 (5)	C12—C13—C14—C15	−1.2 (6)
C10—N4—C16—C15	−179.4 (4)	Mn1—O5—C17—C18	−40.2 (6)
C10—N4—C16—C11	1.5 (4)	Mn1—O6—C19—C20	45.1 (7)

### Hydrogen-bond geometry (Å, º)

**Table:**

*D*—H···*A*	*D*—H	H···*A*	*D*···*A*	*D*—H···*A*
N2—H2···O4^i^	0.86	1.96	2.766 (3)	155
N4—H4···O2^ii^	0.86	1.97	2.786 (3)	158
O5—H5*A*···O3^iii^	0.85	1.89	2.710 (3)	161
O6—H6*A*···O1^iv^	0.85	1.88	2.692 (3)	158

Symmetry codes: (i) *x*+1, *y*−1, *z*; (ii) *x*−1, *y*+1, *z*; (iii) *x*+1, *y*, *z*; (iv) *x*−1, *y*, *z*.
